# A Social Identity Approach to Understanding Responses to Child Sexual Abuse Allegations

**DOI:** 10.1371/journal.pone.0153205

**Published:** 2016-04-25

**Authors:** Kiara Minto, Matthew J. Hornsey, Nicole Gillespie, Karen Healy, Jolanda Jetten

**Affiliations:** 1 School of Psychology, The University of Queensland, Brisbane, Australia; 2 UQ Business School, The University of Queensland, Brisbane, Australia; 3 School of Nursing, Midwifery and Social Work, The University of Queensland, Brisbane, Australia; Bournemouth University, UNITED KINGDOM

## Abstract

Two studies investigated the role of group allegiances in contributing to the failure of institutions to appropriately respond to allegations of child sexual abuse. In Study 1, 601 participants read a news article detailing an allegation of child sexual abuse against a Catholic Priest. Catholics were more protective of the accused–and more skeptical of the accuser—than other participants, an effect that was particularly pronounced among strongly identified Catholics. In Study 2 (*N* = 404), the tendency for Catholics to be more protective of the accused and more skeptical of the accuser than non-Catholics was replicated. Moreover, these effects held independently of the objective likelihood that the accused was guilty. Overall, the data show that group loyalties provide a psychological motivation to disbelieve child abuse allegations. Furthermore, the people for whom this motivation is strongest are also the people who are most likely to be responsible for receiving and investigating allegations: highly identified ingroup members. The findings highlight the psychological mechanisms that may limit the ability of senior Church figures to conduct impartial investigations into allegations of child abuse within the Church.

## Introduction

International reports have highlighted the numerous failures of institutions to protect from harm the vulnerable children entrusted to their care [1,2]. Religious, educational, charitable, sporting, health and correctional institutions have all been subject to inquiries due to their failure to uphold a duty of care to children in their charge. Furthermore, many cases of child sexual abuse go unreported with some research suggesting that 40% of mandated reporters had at some time failed to report suspected abuse [3]. The fact that members of some institutions went to significant lengths to protect the reputation of the institution (and by extension the perpetrators) is of particular concern.

Although there is speculation about the social and personal factors that may contribute to the lack of willingness to examine allegations, the research is predominantly qualitative and anecdotal. For example, interview data suggest that the failure to report suspected child abuse is linked to concerns that the intervention will result in the withdrawal of the child from the family unit [4,5]; negative perceptions of child protection services [6]; and uncertainty about whether the abuse is real [7]. More recently, Staller [8] used qualitative techniques to critically analyze three case studies of institutional child sexual abuse. Factors identified as influencing reporting behaviors include confidentiality mandates; unequal power of victims, offenders and prospective reporters; and strong personal, professional and institutional loyalties.

In this paper, we focus on the influence of professional and institutional loyalties on responses to allegations of child sexual abuse. We extend prior research by experimentally testing a formal theoretical framework: namely, a social identity perspective on responding to deviance. Social identity theory [9,10] makes the case that our self-image reflects not just our *personal identities*–the idiosyncratic traits, behaviors and memories that make us distinct as individuals–but also our *social identities*, which refers to that aspect of our self-definition that we draw from our group memberships. When a particular social identity is salient (e.g., our gender, profession, or religion), our sense of self is influenced by the normative attitudes and behaviors of that identity; particularly so if the group is important to you (i.e., you’re a “high identifier”). Importantly for the current research question, social identity theory also works from the premise that people are motivated to see their group membership through a positive lens: so just as we are motivated to think that we are good people, we are motivated to believe that the groups to which we belong are good groups, worthy of pride.

This theory presents two competing possibilities about the way group allegiances might influence responses to allegations of child sexual abuse, as described below. In this paper, we report two experiments designed to referee between these competing predictions. Specifically, the current studies experimentally examine–for the first time–the role that group allegiance has on how group members respond to allegations of child abuse. In doing so, the data speak to a broader theoretical question about whether—and to what extent–ingroup members protect those within their ranks who have been accused of extreme transgressions.

### Responding to deviance from a social identity perspective

Social psychology has traditionally defined *deviance* as the violation of a group norm or societal standard [11]. When faced with deviance, people can reject, tolerate or attempt to correct the deviant individual. A common sense perspective suggests that severe transgressions are likely to result in rejection of the deviant through derogation, ostracism or expulsion from the group. Indeed, in light of research showing that moral integrity is highly important for a group’s self-concept [12,13], deviance that violates the moral norms of both the group and society is particularly likely to elicit negative reactions from others.

Furthermore, a significant body of research drawing on social identity theory suggests that the rejection of the deviant would be particularly pronounced among ingroup members—the so-called ‘black sheep effect’ [14,15]. The especially harsh derogation of ingroup deviants has been traced back to several psychological processes. Some of these–for example the reasoning that ingroup deviants threaten the distinctiveness of one’s group relative to relevant outgroups [16,17]–are not particularly relevant to the child abuse context and are raised here for the sake of thoroughness. But there are other motivations that are highly pertinent to the child abuse context. Primary among these is the fact that ingroup deviants threaten the positive self-concept that people draw from their group membership, and rejection of that deviant is a way of maintaining or restoring the ingroup’s pride and reputation [14,15,18]. Rejection of deviant ingroup members also serves a function in terms of collective self-definition, clarifying and dignifying the norms that have been challenged by the deviant (see [11] for a review). Both these motives might be expected to be particularly relevant for people who are highly invested in the group, which helps explain why the derogation of ingroup deviants tends to be more pronounced among high identifiers [14,18].

In the context of allegations of child sexual abuse, however, there are reasons to believe that the traditional ‘black sheep effect’—whereby ingroup members downgrade deviants more than outgroup members do—may not emerge (or may even be reversed). First, unless a perpetrator confesses, allegations usually involve one person’s word being pitched against another, and so the guilt of the accused is ambiguous. In the experimental paradigms that have been used so far this is rarely the case: in the social psychology literature deviance has typically been examined in contexts where the deviance was unambiguous. An exception was a study by Van Prooijen [19], who asked Dutch participants to read an allegation of illegal ticket sales for a football game between the Netherlands and Germany. The nationality of the ticket seller was manipulated to be either Dutch (ingroup) or German (outgroup), and participants were exposed to additional text in which the guilt of the suspect was described as either certain or ambiguous. When guilt was certain, participants judged ingroup offenders more harshly than outgroup offenders, the traditional pattern. But when guilt was uncertain, the reverse was true: participants were more lenient towards ingroup offenders than outgroup offenders.

Another factor that moderates how people judge ingroup deviance is the extent to which they see moral superiority as a defining attribute of the group. Iyer, Jetten, and Haslam [20] exposed participants to examples of ingroup deviance; for example reports of prisoner abuses and torture perpetrated by British soldiers. Participants who came into the experiment with stronger views about the moral integrity of the British army were *less* likely to derogate the soldiers’ actions, and particularly when they were highly identified with being British. The authors concluded that when the group is believed to be basically good and moral, a strong commitment to the group blinds group members to its faults. Echoes of this conclusion have been found in anecdotal reports of failures to report child sexual abuse against Catholic clergy. Doyle [21], for example, suggests that there exists within society a pervasive belief that religious clerics are ultimately good people whose actions should not be subject to the same questions as lay people. The high moral standards the group has set for itself makes it harder to believe that ingroup members would engage in violations of that moral norm [22].

The psychological motivation to believe in the innocence of the accused might be particularly pronounced when the psychological consequences of presuming guilt are severe. For example, the Catholic Church places a high emphasis on morality and its capacity to provide moral guidance. Accordingly, threats to the perceived morality of the group would be extremely distressing for Catholic individuals and would likely have a relatively high impact on members’ self-concept. The threat to the status of the Catholic Church is also significant: as representatives of a highly ‘moral’ institution, the community places significant trust in Catholic officials. Public awareness of moral transgressions within the Catholic Church would threaten this trust and consequently the status of the Catholic Church as a group. In this context, recognizing and punishing deviance is a painful and distal way to protect the integrity and purity of the group’s image: a less painful and more proximal way to protect one’s collective self-image is to will oneself into believing that the accusations are false. All these psychological mechanisms suggest that, rather than being especially harsh toward those accused of child sexual abuse within their ranks, people may be more likely to *defend* the honor of other ingroup members.

### The current research

The current studies sought to ascertain the social identity factors that contribute to the failure of institutions to appropriately respond to allegations of child sexual abuse. To examine this, we focused on an allegation of child sexual abuse against a Catholic Church Priest. In Study 1, we examined how different people responded to the allegations depending on their own group loyalties (i.e., as a Catholic, as a non-Catholic Christian or as a non-Christian). Study 2 replicated the design, and in addition examined whether the objective likelihood that the accused priest was guilty moderated the effects of group membership on responses to the accused.

## Study 1

Participants were exposed to text paraphrased from a real news article in which an allegation of child sexual abuse was made against a Priest [23]. The article clearly detailed an allegation rather than a conviction of child sexual abuse, and the arguments of the alleged victim and the Priest were both presented to participants. This ambiguity of guilt reflects the reality of many child sexual abuse situations. Participants comprised Catholics (ingroup), non-Catholic Christians (outgroup members who share a superordinate group membership with Catholics), and non-Christians (outgroup).

Based on the existing literature, one might expect that derogation of the accused would be more pronounced among ingroup members (i.e., Catholic participants) and particularly so among highly identified ingroup members. But, as discussed earlier, there are psychological and contextual factors surrounding child abuse allegations against priests that might suggest an ingroup favoritism effect, such that Catholics are more protective of the accused than are non-Christians (with non-Catholic Christians in between). Specifically, one might expect that ingroup members would defend the integrity of the priest–and question the integrity of the accuser–more so than other participants, and especially when they are strongly identified with their religion.

### Method

#### Participants and Design

Participants were 601 American residents (55% female) aged from 18 to 84 (*M* = 46.30 years). Participants were recruited through the Survey Sampling International (SSI) database and received internet store credit as compensation for their participation. Depending on their self-nominated religious affiliation, participants were categorized as Catholic (26.5%), non-Catholic Christian (53.2%), or non-Christian (20.3%). The other independent variable–religious identification–was a measured variable.

#### Ethics Statement

Both studies in this paper obtained ethical clearance from the Behavioral and Social Sciences Ethical Review Committee at the University of Queensland. Before completing the questionnaire, participants were informed about the aims of the study, and provided their written consent. To limit the potential for distress, participants were advised, prior to the study, of the specific issues addressed in the study. This allowed participants to refrain from participating in the study if they believed it would upset them. They were also made aware of counselling services and provided with contact information in the debriefing sheet.

#### Measures and Procedure

Participants were told that the purpose of the study was “to assess attitudes to alleged moral transgressions in institutions or organisations usually viewed as highly moral”. They were informed in advance that the chosen moral transgression would be an allegation of child sexual abuse and the chosen institution was the Catholic Church. Participants’ right to withdraw at any time without penalty was emphasized. We felt there was an ethical imperative to be up-front about the topic of study, although doing so raises the possibility of self-selection bias such that those who chose to participate were particularly interested in this topic. We acknowledge that *dis*interested participants may be under-represented, and that this represents a caveat to our conclusions.

Participants first recorded their age, sex, race/ethnicity, nationality and religious affiliation. Religious affiliation response options were: 1 Atheist/Agnostic, 2 Catholic, 3 Baptist, 4 Lutheran, 5 Methodist, 6 Church of Jesus Christ of Latter-day Saints, 7 Anglican, 8 Presbyterian, 9 Evangelical, 10 Protestant other, 11 Christian Other, 12 Non-Christian Other (e.g., Judaism, Buddhism, Islam, Hinduism) and 13 Non-Religious Other (e.g., Animism). Based on their responses, participants were categorized as Catholic (those who selected response 2), non-Catholic Christian (those who responded 3–11) or non-Christian (those who responded 1, 12, or 13).

After completing demographic information, participants recorded their strength of religious identification using the ingroup identification scale by Leach and colleagues [24]. Participants responded to statements assessing each of five sub-factors: *solidarity* (e.g., “I feel solidarity with ____ people”), *satisfaction* (e.g., “I am glad to be a ____ person”), *centrality* (e.g., “I often think about the fact that I am a ____ person”), *individual self-stereotyping* (e.g., “I have a lot in common with the average ____ person”), and *ingroup homogeneity* (e.g., “____ people have a lot in common with each other”). Participants were instructed to insert their religious affiliation in the place of the ingroup, and responded using a 7-point likert scale (1 *Strongly disagree* to 7 *Strongly agree*). In accordance with common practice, these sub-factors were collapsed into a single, 14-item identification scale (α = .96).

**Transgression allegation:** The transgression allegation was paraphrased from an actual news article detailing an allegation of child sexual abuse against a Philadelphian Priest [23]. The presentation of both the allegation and the Priest’s defence in the article was designed to ensure that the Priest’s guilt was uncertain. The text read as follows:

A 26 year old man has accused a Catholic Priest of sexually abusing him when he was 10 years old. The boy’s mother sought counselling from the priest due to her belief that her son was homosexual. The boy has claimed that the priest gained his trust by providing treats and free run of his house.

On the day of the alleged assault, the boy visited the priest for cookies after a church service. The priest reportedly brought the boy up to his bedroom, removed both their clothes, fondled him and attempted to initiate oral sex. After the alleged assault, the boy claims that the Priest gave him repeated warnings regarding the sinful nature of homosexuality, suggesting that the assault was a punishment for his sin.

The Priest's defence attorney has argued that the case is suspect because the accuser is the only apparent victim of McIntyre. The defence attorney suggests that if the Priest were guilty, there would surely be other victims. He further argues that the accuser's self-confessed past use of illegal drugs may have impaired his recollection of the alleged assault.

After reading the article, participants completed the dependent measures (described below).

**Credibility of alleged offender and alleged victim:** Using a 7-point likert scale (1 = *not at all*, 7 = *extremely*), participants indicated the extent to which they felt that the Priest’s defence was honest, plausible, manipulative, convincing, insincere, trustworthy, and dishonest. Participants then responded to the same items to indicate the extent that the items reflected their perception of the alleged victim. Negatively worded items were reversed and items were averaged to form scales of offender (α = .83) and victim (α = .85) credibility.

**Skepticism about the allegation:** Skepticism about the allegation was measured using 3 items (α = .76) which allowed participants to express their beliefs regarding the validity of the allegation in the text and similar allegations in general (e.g., “Accusations like this are often made up or exaggerated”; “I find it difficult to believe that a religious person could commit a moral transgression like child sexual abuse”; 1 = *strongly disagree* to 7 = *strongly agree*).

### Results and Discussion

Means and correlations among variables are summarized in Table 1. Raw data can be accessed on request. As can be seen there were moderate inter-correlations among the variables. Inspection of the means reveals that participants were more inclined to believe the accuser rather than the accused, but not overwhelmingly so. Ratings of offender credibility and skepticism were modestly below the mid-point, whereas ratings of victim credibility were modestly above the mid-point.

**Table 1:** Intercorrelations Among Variables: Study 1.

	Mean (SD)	2	3	4
1. Religious identification	4.95 (1.22)	.10*	.05	.17***
2. Victim credibility	4.92 (1.07)	-	-.60***	-.35***
3. Offender credibility	3.23 (1.21)	-	-	.40***
4. Skepticism about allegation	3.19 (1.46)	-	-	-

Note.

* *p* < .05

*** *p* < .001. All constructs were measured using 1–7 scales.

The hypotheses were tested in two sets of analyses. In the first set of analyses, univariate ANOVAs were conducted comparing Catholics, non-Catholic Christians, and non-religious participants on the dependent variables. Significant effects were followed up with Duncan’s post hoc tests (see Table 2 for a summary). In the second set of analyses, regressions were conducted to determine whether religious identification moderated the effect of religious group on the dependent variables. Non-Christian participants were excluded from the latter analyses because the presence of many atheists and agnostics in the group rendered the religious identification measure inappropriate.

**Table 2:** Responses to the Allegations as a Function of Participant Religion: Study 1.

Variable	Catholics Mean (SD)	Non-Catholic Christians Mean (SD)	Non-Christians Mean (SD)
Offender credibility	3.41_*a*_ (1.10)	3.26_*a*_ (1.21)	2.91_*b*_ (1.29)
Victim credibility	4.76_*a*_ 0.98)	4.94_*ab*_ (1.08)	5.05_*b*_ (1.13)
Skepticism about allegation	3.60_*a*_ (1.45)	3.19_*b*_ (1.42)	2.67_*c*_ (1.44)

*Note*. Within each variable, means with different subscripts significantly differ from each other.

#### Effects of Participant Religious Group

As can be seen in Table 2, there was a significant effect of religious group on ratings of offender credibility, *F*(2,598) = 6.23, *p* = .002, η^2^ = .020, such that Catholics and non-Catholic Christians both rated the Priest as more credible than non-Christians (there was no significant difference between Catholics and non-Catholic Christians). Religious group also had a marginal effect on participants’ ratings of victim credibility, *F*(2,598) = 2.81, *p* = .061, η^2^ = .010, with Catholics rating the alleged victim as significantly less credible than non-Christians (ratings of non-Catholic Christians did not significantly differ from either group). In sum, Catholics were more protective of the priest–and less supportive of the accuser–than non-Christians. Catholic participants were also significantly more skeptical of the allegation than were non-Catholic Christians, who in turn were significantly more skeptical than non-Christians, *F*(2,591) = 14.39, *p* < .001, η^2^ = .046.

#### Moderating Effects of Identification

To test the moderating role of identification, we conducted regression analyses using Hayes’ [25] PROCESS computational model (Model 1 testing interactions between a continuous and a dichotomous variable). Bootstrapping of 5,000 samples was used, with 95% confidence intervals. For these analyses, identification was mean-centered and religious group was coded such that Catholic = 0 and non-Catholic Christians = 1. Note that the *b* statistics reported throughout are *un*standardized coefficients.

**Fig 1:**
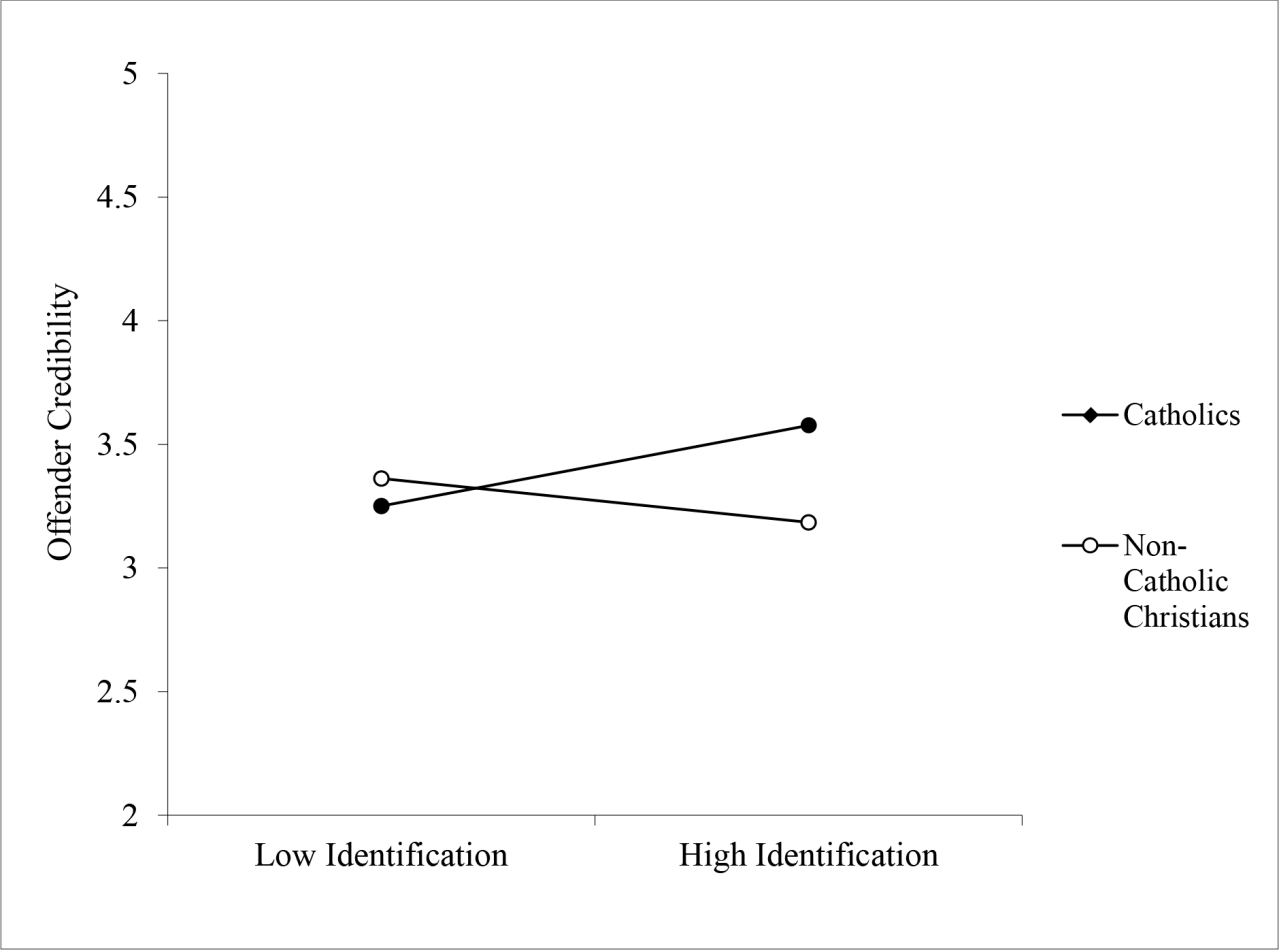
Effect of Religious Identification on Ratings of the Alleged Offender’s Credibility as a Function of Participant Religion (Study 1).

**Offender credibility:** As predicted, a significant interaction between religious group and religious identification emerged on credibility ratings of the Priest, *b* = -0.23, CI_95_[-.434, -.020], *p* = .032 (see Fig 1). Catholic participants viewed the Priest as significantly more credible than non-Catholic Christians when they were high identifiers, *b* = -0.39, CI_95_[-.715, -.068], *p* = .018, but not when they were low identifiers, *b* = 0.10, CI_95_[-.211, .407], *p* = .535. Examining the simple slopes another way, there was a marginally significant positive relationship between identification and offender credibility among Catholic participants, *b* = 0.15, CI_95_[-.021, .315], *p* = .086, but among non-Catholic Christians the relationship was non-significant and in the other direction, *b* = -0.08, CI_95_[-.201, .041], *p* = .195.

**Fig 2:**
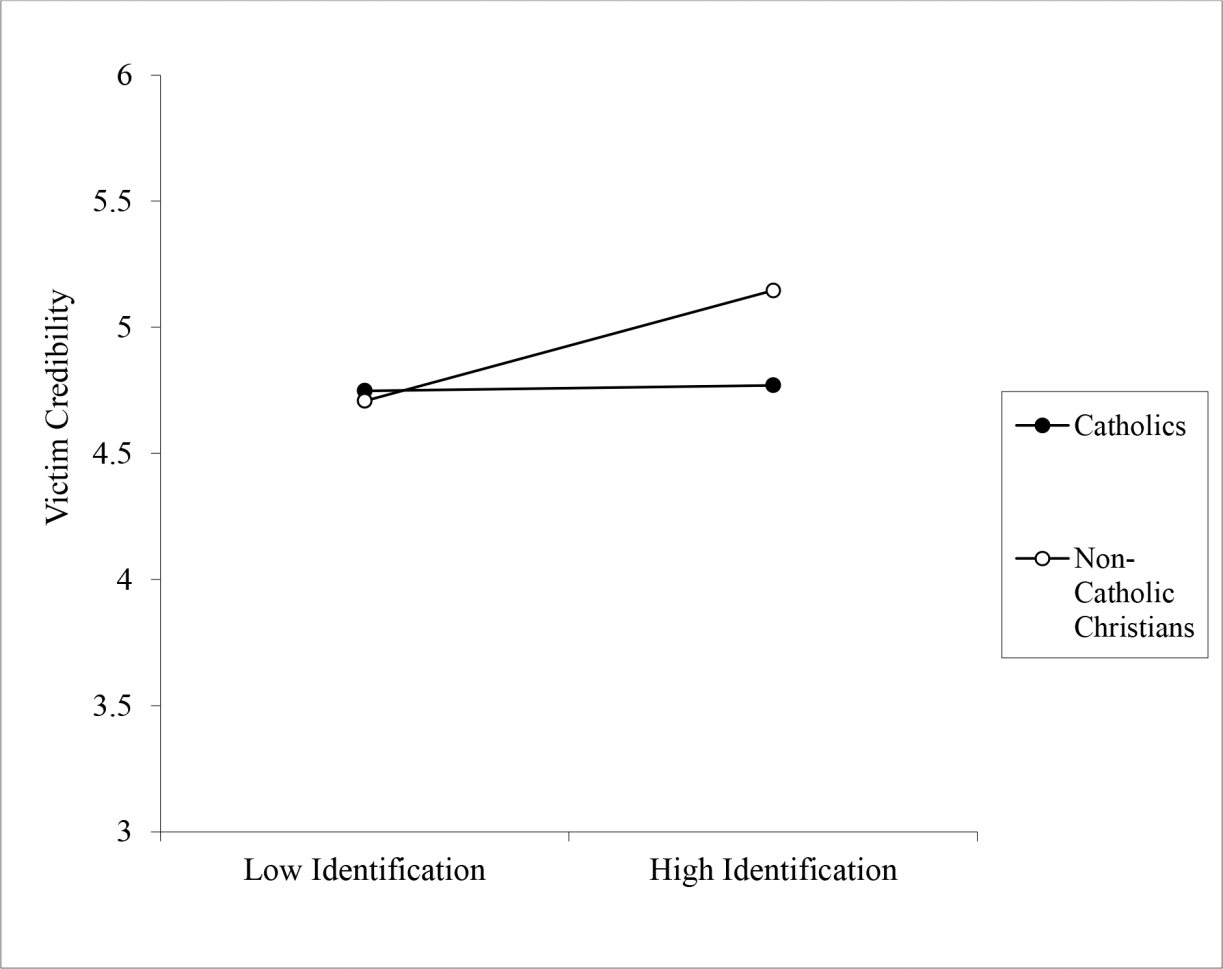
Effect of Religious Identification on Ratings of the Alleged Victim’s Credibility as a Function of Participant Religion (Study 1).

**Victim credibility:** A significant interaction also emerged between religious group and religious identification on the perceived credibility of the victim, *b* = 0.19, CI_95_[.003, .371], *p* = .046 (see Fig 2). Among high identifiers, non-Catholic Christians rated the alleged victim as significantly more credible than Catholics, *b* = 0.37, CI_95_[.087, .663], *p* = .011. This effect was not apparent among low identifiers, however, *b* = -0.03, CI_95_[-.303, .247], *p* = .840. When examining the simple slopes another way, it can be seen that there was a strong positive relationship between identification and victim credibility among non-Catholic Christians, *b* = 0.20, CI_95_[.089, .305], *p* < .001, but among Catholics the relationship was non-significant, *b* = 0.01, CI_95_[-.139, .159], *p* = .896.

**Fig 3:**
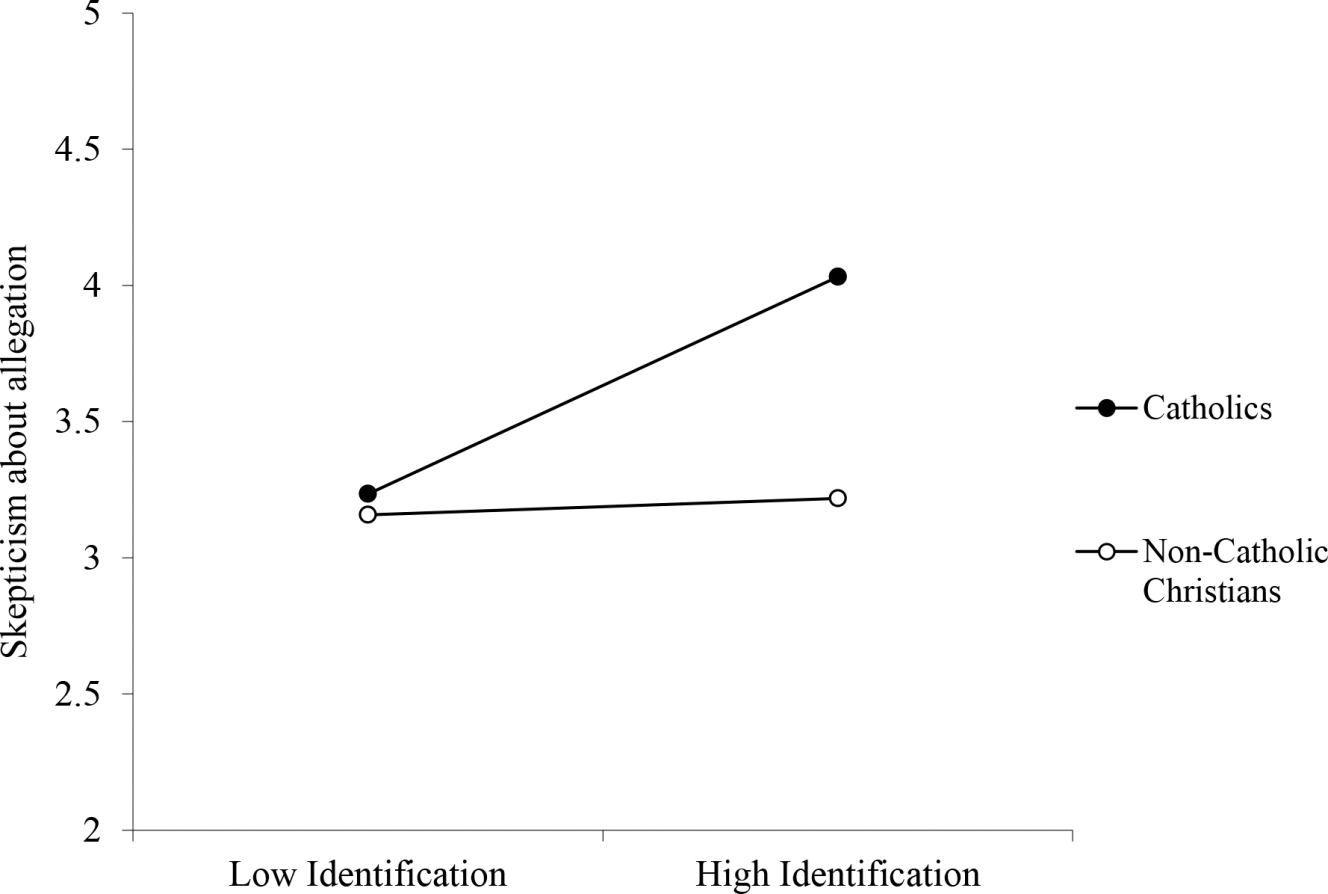
Effect of Religious Identification on Skepticism About the Allegation as a Function of Participant Religion (Study 1).

**Skepticism about allegation:** Significant main effects of religious group and religious identification were qualified by a significant interaction, *b* = -0.33, CI_95_[-.586, -.079], *p* = .010 (See Fig 3). At high levels of identification, Catholics were significantly more skeptical of the allegation than non-Catholic Christians, *b* = -0.81, CI_95_[-1.206, -.412], *p* < .001. At low levels of identification, however, this effect disappeared, *b* = -0.10, CI_95_[-.475, .279], *p* = .609. Similarly, Catholic participants expressed more skepticism about the allegation the more strongly they identified with their religion, *b* = 0.36, CI_95_[.152, .566], *p* < .001, but this was not the case for non-Catholic Christians, *b* = 0.03, CI_95_[-.119, .173], *p* = .717.

In sum, traditional research on deviance has tended to show that transgressions are more likely to be downgraded by ingroup members than by outgroup members. But in the context of child sexual abuse allegations within the Catholic Church, where guilt was implied but ambiguous, this effect did not emerge. Instead, it was Catholic participants who were *particularly* likely to defend the credibility of the accused, and to doubt the credibility of the accuser. Furthermore, these effects were evident exclusively among those for whom religion was an important and central part of their self-concept.

## Study 2

In Study 1 the participants were presented with the accusations much as they might be in a neutral reporting of a criminal trial; as an allegation contextualized by victim testimony, circumstantial evidence, and a defence from the accused. There is rarely “smoking gun” evidence to substantiate child abuse allegations, and the accused rarely spontaneously confess. As such, observers must decide who they trust: the accuser or the accused. In the context of this ambiguity, there is room for highly invested ingroup members to protect the integrity and reputation of their group by placing their faith in the accused and casting doubt on the accuser.

In Study 2, we examined what might happen if the circumstantial case for the guilt of the accused was relatively high. Earlier, we discussed how the ambiguity around an offence can dramatically change responses to deviance: Van Prooijen [19] found an ingroup-favoritism effect when there was ambiguity around a minor criminal act, but when this ambiguity was removed the effect reversed. Similarly, it could be that the relative tendency for Catholics to defend the integrity of an accused priest might reverse if the circumstantial case around the allegation was strong.

We examined this question using the same case study used in Study 1. Because of the sensitive nature of the topic we did not want to use deception in our manipulations. Instead, we selectively presented details around a real child sexual abuse allegation, and framed it in such a way that made the case against the accused priest seem more or less certain. Consistent with Study 1, we expected that in the low guilt-certainty condition Catholics would rate the offender as higher in credibility than would other participants, and that they would rate the credibility of the victim and his accusations as relatively low. Based on Van Prooijen’s [19] studies, we expected that this ingroup favoritism would be weaker or eliminated altogether in the high guilt-certainty condition. Drawing on social identity reasoning, all effects were expected to be stronger the more participants identified with their religion.

### Method

#### Participants and Design

Four hundred and four U.S. residents (56.4% male; *M* = 34.86 years) were recruited through the SSI database. Participants were randomly assigned to the high or low certainty of guilt condition. As in Study 1, participants were categorized as Catholics (*n* = 120), non-Catholic Christians (*n* = 141) or non-Christians (*n* = 143) depending on their self-reported religious affiliation.

#### Measures and Procedure

Religious identification was measured using the same scale used in Study 1 (α = .95).

**Manipulation of guilt certainty:** Participants read the allegation information in the first two paragraphs of the text used in Study 1. Additional text was then included to manipulate the likelihood that participants would see the accused as guilty. Participants in the high guilt certainty condition read this additional text:

The priest has denied that he is guilty. But the archdiocese of the area has made a statement specifying that he and the church will not be involved in the priest's defense. These are not the first child sex abuse charges the accused is facing. He was suspended previously for inappropriate conduct with children separate to the current victim's accusations. A criminal trial is currently underway.

Participants in the low guilt certainty condition read this additional text:

The priest's defense attorney has argued that the case is suspect because the accuser is the only apparent victim of the defendant. The defense attorney suggests that if the priest were guilty, there would surely be other victims. He further argues that the accuser's self-confessed past use of illegal drugs may have impaired his recollection of the alleged assault. In the recently completed trial, the jury decided that there was not enough evidence to warrant a guilty verdict.

Measures of *victim credibility* (α = .90), *offender credibility* (α = .88) and *skepticism about the allegation* (α = .73) were the same as those used in Study 1. In addition, a *perceived guilt* scale was included to assess the success of the manipulation. Participants completed five items assessing the extent to which participants believed the alleged offender to be guilty of the accusations of child sexual abuse, for example “I think the priest is guilty of child sex abuse”; “I believe the priest 'groomed' the boy for sexual abuse”; and “I believe that the accuser misinterpreted and exaggerated the actions of the priest” (reversed; α = .93). All items in this study were assessed using 7-point likert scales ranging from 1 (*strongly disagree*) to 7 (*strongly agree*). Raw data are available upon request.

### Results and Discussion

As in Study 1, we first conducted ANOVAs comparing the three religious groups (see Table 3 for a summary of means) before doing follow-up regressions on the two Christian groups to test the moderating role of religious identification.

**Table 3:** Responses to the Allegations as a Function of Participant Religion: Study 2.

Variable	Catholics Mean (SD)	Non-Catholic Christians Mean (SD)	Non-Christians Mean (SD)
Offender credibility	3.12_*a*_ (1.28)	2.75_*b*_ (1.31)	2.49_*b*_ (1.22)
Victim credibility	5.25_*a*_ (1.17)	5.51_*b*_ (1.12)	5.63_*b*_ (0.97)
Skepticism about allegation	3.17_*a*_ (1.43)	2.73_*b*_ (1.45)	2.20_*c*_ (1.12)
Perceived guilt	5.14_*a*_ (1.31)	5.36_*ab*_ (1.35)	5.57_*b*_ (1.12)

*Note*. Within each variable, means with different subscripts differ significantly from each other.

#### Effects of Guilt Certainty and Religious Group

**Perceived guilt:** Consistent with the manipulation, participants in the high certainty of guilt condition (*M* = 5.71, *SD* = 1.17) rated the alleged offender as significantly more guilty than participants in the low certainty of guilt condition (*M* = 5.03, *SD* = 1.29), *F*(1,395) = 31.36, *p* < .001, η^2^ = .07. There was also a main effect of religious group, *F*(2,395) = 4.32, *p* = .014, η^2^ = .02. Catholics’ mean ratings of the offender’s guilt were significantly lower than non-Christians’ ratings of the offender’s guilt. Non-Catholic Christians’ ratings of offender guilt did not differ significantly from either Catholics or non-Christians. There was no interaction of guilt certainty and religious group, *F*(2,395) = 0.33, *p* = .718, η^2^ = .00.

**Offender credibility:** Catholics rated the alleged offender as more credible than did non-Catholic Christians and non-Christians (the latter two conditions did not differ from each other), *F*(2,397) = 8.96, *p* < .001, η^2^ = .04. Participants in the high guilt-certainty condition rated the alleged offender as less credible than participants in the low certainty condition, *F*(1,397) = 24.96, *p* < .001, η^2^ = .06, but again, guilt certainty did not interact with religious affiliation, *F*(2,397) = 0.16 *p* = .849, η^2^ = .00.

**Victim credibility:** Catholics rated the alleged victim as less credible than did non-Catholic Christians and non-Christians (the latter two conditions did not differ from each other), *F*(2,397) = 4.79, *p* = .009, η^2^ = .02. Perhaps unsurprisingly, participants in the high guilt-certainty condition rated the alleged victim as more credible than did participants in the low certainty condition, *F*(1,397) = 31.24, *p* < .001, η^2^ = .07, but the guilt-certainty manipulation did not interact with religious affiliation, *F*(2,397) = 0.10, *p* = .904, η^2^ = .00.

**Skepticism about the allegation:** Catholics were significantly more skeptical of the allegation than non-Catholic Christians who in turn were significantly more skeptical than non-Christians, *F*(2,396) = 17.05, *p* < .001, η^2^ = .08. Certainty of guilt condition had no effect on participants’ skepticism about the allegation, *F*(1,396) = 0.03, *p* = .853, η^2^ = .00, and there was no interaction, *F*(2,396) = 0.30, *p* = .738, η^2^ = .00.

#### Moderation by Identification

Regression analyses were conducted to determine whether certainty of guilt condition or religious identification moderated the effect of religious group membership on the dependent variables. As for Study 1, non-Christian participants were excluded from the analyses. We do not report the main effects of guilt and religious group because they duplicate the effects reported in the ANOVAs above.

The only significant effects of identification were the predicted interactions between identification and religious group on offender credibility, *b* = 0.35, CI_95_[-.622, -.077], *p* = .012, and skepticism about the allegation, *b* = -0.33, CI_95_[-.633, -.017], *p* = .039. At high levels of identification, Catholics rated the alleged offender as more credible than did non-Catholic Christians, *b* = -0.86, CI_95_[-1.418,- .295], *p* = .003, and Catholics were more skeptical of the allegation than were non-Catholic Christians, *b* = -1.08, CI_95_[-1.716, -.449], *p* = .001. Among low identifiers, these effects disappeared (*p*s > .28).

There was also a marginally significant interaction of religious group and religious identification on guilt ratings, *b* = 0.25, CI_95_[-.027, .528], *p* = .077. At high levels of identification, non-Catholic Christians were more likely to believe the priest was guilty than were Catholics, *b* = 0.60, CI_95_[.028, 1.171], *p* = .040, an effect that did not emerge among low identifiers, *b* = 0.02, CI_95_[-.523, .561], *p* = .95.

The predicted identification by religious group interaction trended on ratings of victim credibility but fell short of significance, *b* = 0.20, CI_95_[-.040, .435], *p* = .102. Furthermore, identification did not feature in any significant 2-way or 3-way interactions with guilt certainty (all *p*s > .55).

In sum, the results replicated those of Study 1: ingroup participants were more likely to defend the integrity of the accused (and to cast doubt on the accuser) than were other participants, an effect that was exclusively driven by high identifiers. Interestingly–and somewhat surprisingly–this effect was not moderated by the subjective level of certainty surrounding the guilt of the accused. The lack of such effects was not due to problems associated with the manipulation: in the high-certainty condition participants were more likely to believe the priest was guilty than in the low-certainty condition, with predictable downstream consequences in terms of ratings of credibility. But this manipulation did not moderate the effects of religious group. The tendency for (highly identified) Catholics to be relatively protective of the accused remained equally strong regardless of the scale of the circumstantial case against him.

## General Discussion

Social psychological research on deviance has shown that group members are especially keen to derogate and reject norm-breakers *within* their ranks. One might expect from this research that Catholic participants would be more motivated than anybody to derogate and exclude church members who face serious and credible allegations of child sexual abuse. But across two studies we found the opposite tendency: Catholic participants were more likely than non-Catholic participants to cast doubt on the credibility of the accusations, and to defend the credibility of the accused.

The results of the current study provide a possible explanation for the failure of senior group members to respond appropriately to allegations of child sexual abuse in the institutional context. Because allegations of child sexual abuse are typically referred to those with the power to deal with the accused appropriately, the recipients of allegations are also likely to be high identifying members of the same institution as the accused. Our data confirm that such highly identified ingroup members are the *least* willing to believe that the accusations are based on fact. This helps to provide psychological explanations for qualitative and anecdotal accounts of senior group members failing to adequately follow up allegations of child sexual abuse within their institution [3,8].

We presume that the underlying motive for these effects is ingroup members’ need to protect and enhance their collective self-image. But this raises the question: Why would our highly identified Catholics protect their group image by defending the accused priest, when they could have chosen to purify the group by derogating and excluding him?

Intuitively, the fact that the deviance is contested–that it was an *allegation* rather than a demonstrable act—would seem to be an important factor that makes this context different from most examined in the existing deviance literature. The non-verifiable nature of the deviance leaves room for the motivated perceiver to defend the group through denial: one can imagine the accuser to be unreliable; the accused to be an innocent victim of a malicious claim. This is a tempting psychological pathway, and is potentially less painful than the alternative, which would be to accept that senior ingroup members are capable of violating sanctified norms and abusing the trust of their community in such a dramatic way.

If the allegations were beyond doubt–if there was documented evidence of the abuse or the priest had confessed–then we believe that our ingroup participants may well have shown heightened derogation of the priest in the manner of the previous literature. But it should be noted that this is rarely the case with sexual abuse, which by its nature is typically conducted in private and shrouded in ambiguity. In many deviance experiments, the events are clear and uncontestable, handed to the participants by an experimenter who has the power to present the facts in a way that eliminates ambiguity and sharpens the boundaries between normative and non-normative behavior. But in real life this is often not the case, and in cases of sexual abuse it is almost never the case: to decide that an ingroup member is guilty, and to excommunicate them from the group as a result, is in itself a leap of faith in the accuser. Furthermore, it is a leap of faith that defies psychological gravity given ingroup members’ understandable desire to believe that the accused is innocent. We believe that this helps explain one of the more surprising aspect of our results; the fact that in Study 2 there were signs of ingroup favoritism *even when* the circumstantial case made against the accused was strong. Study 2 tells us that, when ingroup members are motivated to defend the honor of the accused, they only need a small sliver of ambiguity to justify doing so.

This motivation to defend the ingroup accused might be exaggerated by the fact that ingroup members–particularly highly identified ones–presumably have internalized a conviction that their group is highly moral, which could introduce another layer of psychological implausibility around the allegation. There is qualitative evidence that some church members are so convinced of the sanctity of their senior officials that they struggle to believe that they could be capable of child abuse [22]. This dovetails with experimental evidence that, when people are convinced of the moral virtues of their group, they are somewhat slower to recognize and condemn moral violations within their group [20].

Another factor that is somewhat specific to this context is the fact that allegations of child sexual abuse within the Catholic Church have been a long-standing source of tension and upset, played out in a highly public series of scandals. The Catholic Church has faced intense criticism of its handling of allegations from many quarters, and for many outsiders the scandals have become a defining flaw of the group culture. For any single incident such as the one described in the current studies, it may feel to Catholics that it is not just the accused priest on trial, but the whole institution. Research on group criticism highlights the tendency for group members to reject even reasonable messages for change when they are voiced by outsiders, and to go into denial about problems that they might otherwise freely acknowledge [26,27]. It could be that allegations of abuse are now viewed through an intergroup prism by some Catholics, reinforcing a “them-against-us” mentality that led to denial and defensiveness, even in the face of what seem on the surface to be credible allegations.

Given this, it remains an open question whether the effects reported here would generalize to contexts of institutional transgressions other than child sex abuse allegations within the Catholic Church. Our analysis above suggests certain variables–for example whether the group is defined by their moral reputation; whether the transgressions are made public; whether the group is part of a protracted ‘us-against-them’ battle to protect its reputation–that might moderate the observed effects. Future research is needed to sharpen this theorizing, and to nuance psychological responses to different types of transgressions in different types of institutions. Future research could also explore how responses might be affected by the group allegiances of the victim. In the current studies, the perpetrator *and* the victim were Catholics, meaning that ingroup loyalties would have been somewhat divided between the accused and the accuser. It could be that the relative protection of the accused among Catholics would be even more pronounced should the victim be an outgroup member.

Finally, it is important to acknowledge that the modal response among the participants across both studies was to believe the accusations and to doubt the credibility of the accused. Even the Catholic participants, on average, were inclined to take the allegations on face value. So what we are witnessing here is different degrees of faith in the accuser, and suspicion of the accused. For those who may fear that their reports of abuses may be met with disbelief, this is an encouraging message from the data.

Furthermore, we hasten to add that the specific incident used in our experimental studies was described as an allegation, and so we do not presume that participants who questioned the veracity of the claims did so inappropriately. We do, however, argue that the current paradigm uncovers a psychological mechanism that may help shed light on why allegations may not be investigated or followed up a vigorously as they should.

In sum, the current studies provide a basis to conclude that social identity factors influence responses to allegations of child sexual abuse in institutional contexts. Group loyalties provide a psychological motivation for some to disbelieve allegations. Furthermore, the people for whom this motivation is strongest are also the people who are most likely responsible for receiving and investigating allegations *and* protecting the group’s reputation: highly identified ingroup members who are invested in the idea that their group has high moral integrity. On the surface this would seem like an unfortunate conflict of psychological interests, and it seems plausible that this contributes to the troubling failure of some institutions to adequately protect the safety of children in their care. Our findings underscore the importance of external governance mechanisms to facilitate the timely, independent review of allegations of misconduct in religious organizations. This provides a mechanism for allegations to be investigated by an independent body, and hence by individuals that are not highly identified with the organization.

## Supporting Information

S1 Dataset Supporting dataset. (SAV)

S2 Dataset Supporting dataset. (SAV)

## References

[1] Australian Institute of Family Studies. Institutional Child Abuse Inquiries 2002–2013. 2014. Available: http://www.aifs.gov.au/institute/pubs/carc/4.html

[2] Historical Institutional Abuse Inquiry–The Background. (2014, January 13). BBC News. Retrieved from http://www.bbc.com/news/uk-northern-ireland-25637486

[3] Zellman GL, Antler S. Reporting of child maltreatment In: Briere J, Berliner L, Bulkley JA, Jenny C, Reid T, editors. The APSAC handbook of child maltreatment. Thousand Oaks, CA: Sage; 1996 pp. 359–381.

[4] Bryant JK. School counselors and child abuse reporting: A national survey. Professional School Counseling. 2009; 12: 333–342. 10.5330/PSC.n.2010-12.333

[5] Crenshaw WB, Lichtenberg JW, Bartell PA. Mental health providers and child sexual abuse: A multivariate analysis of the decision to report. Journal of Child Sexual Abuse. 1994; 2: 19–42. 10.1300/J070v02n04_02

[6] Vulliamy AP, Sullivan R. Reporting child abuse: Pediatricians’ experiences with the child protection system. Child Abuse & Neglect. 2000; 24: 1461–1470. 10.1016/S0145-2134(00)00199-X 11128177

[7] Eisbach SS, Driessnack M. Am I sure I want to go down this road? Hesitations in the reporting of child maltreatment by nurses. Journal for Specialists in Pediatric Nursing. 2010; 15: 317–323. 10.1111/j.1744-6155.2010.00259.x 20880280

[8] Staller KM. Missing pieces, repetitive practices: Child sexual exploitation and institutional settings. Cultural Studies–Critical Methodologies. 2012; 12: 274–278. doi:10.1177/1532708612446420

[9] Tajfel H, Turner JC. The social identity theory of intergroup behavior In: Worchel S, Austin E, editors. Psychology of intergroup relations. Chicago: Nelson-Hall; 1986 pp. 7–24.

[10] Hornsey M J. Social identity theory and self-categorization theory: A historical review. Social and Personality Psychology Compass. 2008; 1: 204–222. 10.1111/j.1751-9004.2007.00066.x

[11] Jetten J, Hornsey MJ. Deviance and dissent in groups. Annual Review of Psychology. 2014; 65: 461–485. 10.1146/annurev-psych-010213-115151 23751035

[12] Leach CW, Ellemers N, Barreto M. Group virtue: The importance of morality (vs. competence and sociability) in the positive evaluation of in-groups. Journal of Personality and Social Psychology. 2007; 93: 234–249. 10.1037/0022-3514.93.2.234 17645397

[13] Pagliaro S, Ellemers N, Barreto M. Sharing moral values: Anticipated ingroup respect as a determinant of adherence to morality-based (but not competence-based) group norms. Personality and Social Psychology Bulletin. 2011; 37: 1117–1129. 10.1177/0146167211406906 21540366

[14] Hutchison P, Abrams D, Gutierrez R, Viki GT. Getting rid of the bad ones: The relationship between group identification, deviant derogation, and identity maintenance. Journal of Experimental Social Psychology. 2008; 44: 874–881. 10.1016/j.jesp.2007.09.001

[15] Marques JM, Paez D. The “black sheep effect”: social categorization, rejection of ingroup deviates, and perception of group variability. European Review of Social Psychology. 1994; 5: 37–68. 10.1080/14792779543000011

[16] Abrams D, Marques JM, Bown N, Henson M. Pro-norm and anti-norm deviance within in-groups and out-groups. Journal of Personality and Social Psychology. 2000; 78: 906–912. 10.1037/0022-3514.78.5.906 10821197

[17] Warner R, Hornsey MJ, Jetten J. Why minority members resent impostors. European Journal of Social Psychology. 2007; 37: 1–18. 10.1002/ejsp.332

[18] Marques JM, Yzerbyt VY, Leyens J. The “black sheep effect”: Extremity of judgments towards ingroup members as a function of group identification. European Journal of Social Psychology. 1988; 18: 1–16. 10.1002/ejsp.2420180102

[19] Van Prooijen J-W. Retributive reactions to suspected offenders: The importance of social categorizations and guilt probability. Personality and Social Psychology Bulletin. 2006; 32: 715–726. 10.1177/0146167205284964 16648197

[20] Iyer A, Jetten J, Haslam SA. Sugaring o’er the devil: Moral superiority and group identification help individuals downplay the implications of ingroup rule-breaking. European Journal of Social Psychology. 2012; 42: 141–149. 10.1002/ejsp.864

[21] Doyle TP. Clericalism: Enabler of clergy sexual abuse. Pastoral Psychology. 2006; 54: 189–213. 10.1007/s11089-006-6323-x

[22] Scheper-Hughes N, Devine J. Priestly celibacy and child sexual abuse. Sexualities. 2003; 6: 15–40. 10.1177/1363460703006001003

[23] Kurvilla C (2014, February 28). Former altar boy reveals grim details of Philadelphia priest’s alleged sex abuse. New York Daily News. Available http://www.nydailynews.com/news/crime/altar-boy-reveals-grim-details-philadelphia-priest-sex-abuse-article-1.1706488 Accessed May 2014.

[24] Leach CW, van Zomeren M., Zebel S, Vliek MLW, Pennekamp SF, Doosje B et al Group-level self-definition and self-investment: A hierarchical (multicomponent) model of in-group identification. Journal of Personality and Social Psychology. 2008; 95: 144–165. 10.1037/0022-3514.95.1.144 18605857

[25] Hayes AF. An introduction to mediation, moderation, and conditional process analysis: A regression-based approach New York, NY: Guilford Press 2013.

[26] Esposo SR, Hornsey MJ, Spoor JR. Shooting the messenger: Outsiders critical of your group are rejected regardless of argument quality. British Journal of Social Psychology. 2013; 52: 386–395. 10.1111/bjso.12024 23316747

[27] Hornsey MJ, Imani A. Criticizing groups from the inside and the outside: An identity perspective on the intergroup sensitivity effect. Personality and Social Psychology Bulletin. 2004; 30: 365–383. 10.1177/0146167203261295 15030626

